# Advancing the match-mismatch framework for large herbivores in the Arctic: Evaluating the evidence for a trophic mismatch in caribou

**DOI:** 10.1371/journal.pone.0171807

**Published:** 2017-02-23

**Authors:** David Gustine, Perry Barboza, Layne Adams, Brad Griffith, Raymond Cameron, Kenneth Whitten

**Affiliations:** 1 U.S. Geological Survey, Alaska Science Center, Anchorage, Alaska, United States of America; 2 Wildlife and Fisheries Science, Texas A&M University, College Station, Texas, United States of America; 3 U.S. Geological Survey, Alaska Cooperative Fish and Wildlife Research Unit, University of Alaska Fairbanks, Fairbanks, Alaska, United States of America; 4 Alaska Department of Fish and Game, Fairbanks, Alaska, United States of America; Universita degli Studi di Sassari, ITALY

## Abstract

Climate-induced shifts in plant phenology may adversely affect animals that cannot or do not shift the timing of their reproductive cycle. The realized effect of potential trophic “mismatches” between a consumer and its food varies with the degree to which species rely on dietary income and stored capital. Large Arctic herbivores rely heavily on maternal capital to reproduce and give birth near the onset of the growing season but are they vulnerable to trophic mismatch? We evaluated the long-term changes in the temperatures and characteristics of the growing seasons (1970–2013), and compared growing conditions and dynamics of forage quality for caribou at peak parturition, peak lactation, and peak forage biomass, and plant senescence between two distinct time periods over 36 years (1977 and 2011–13). Despite advanced thaw dates (7−12 days earlier), increased growing season lengths (15−21 days longer), and consistent parturition dates, we found no decline in forage quality and therefore no evidence within this dataset for a trophic mismatch at peak parturition or peak lactation from 1977 to 2011–13. In Arctic ungulates that use stored capital for reproduction, reproductive demands are largely met by body stores deposited in the previous summer and autumn, which reduces potential adverse effects of any mismatch between food availability and timing of parturition. Climate-induced effects on forages growing in the summer and autumn ranges, however, do correspond with the demands of female caribou and their offspring to gain mass for the next reproductive cycle and winter. Therefore, we suggest the window of time to examine the match-mismatch framework in Arctic ungulates is not at parturition but in late summer-autumn, where the multiplier effects of small changes in forage quality are amplified by forage abundance, peak forage intake, and resultant mass gains in mother-offspring pairs.

## Introduction

The strongest evidence on the climate-mediated effects of phenological mismatches on animals has primarily come from species that rely upon current food resources (i.e., dietary income) to complete reproduction [1, 2–4]. Small animals are vulnerable to failures in income from food resources that coincide with their commitments to breeding [5, 6]. Conversely, large animals are more vulnerable to failures of prior income in stored capital that is used to complete reproduction [7, 8]. For large animals, phenological or trophic mismatches emphasize income failures when capital is already low and may be more vulnerable, to varying degrees, to capital failures because their reproductive demands usually exceed current income [9]. Thus, body size affects the strength of the relationship between current environmental supply and animal demand that ultimately affects the response of a species to a particular change in climate [10].

Climate warming has been associated with a shift in the phenology of forage production and quality for large herbivores. In tropical savannas, warming has shifted the timing, duration, and extent of the annual rainy and dry seasons and their relative influences to primary productivity [11, 12]. In the Arctic, warmer temperatures and changes to hydrologic regimes have advanced the onset and extended the duration of the growing season [13], yet, the magnitude of the vegetative responses has varied [14, 15]. Similarly, population responses by large herbivores may be mediated, in part, by their capacity to “match” the shifting availability of forage resources with nutritional demands of reproduction [7] and (or) their capability to establish nutritional stores essential for survival and early reproduction [16].

Climate-induced vegetative changes throughout the growing season have demonstrated strong trends, yet there have been no documented trends in reproductive phenology of northern ungulates such as caribou, reindeer (*Rangifer tarandus*), and muskoxen (*Ovibos moschatus*). Reproductive phenology is closely related to photoperiod, the timing and duration of the growing season, and the duration and severity of winter [17, 18]. Births are generally synchronous by species during April to June [19], however, the actual timing of births relative to the onset of the growing season varies with species and degree of reliance on stored capital [20]. Parturition precedes or coincides with the onset of the growing season and is timed to maximize the period of peak nutrient availability (nutrient concentration x abundance x digestibility) to mother-offspring pairs [21]. The trophic mismatch hypothesis for large herbivores states that earlier green-up will shift peak nutrient availability away from peak nutritional demand leading to lower productivity [22]. However, this hypothesized mechanism for evaluating the influences of climate change on reproductive success in northern ungulates has not been evaluated consistently. For example, in western Greenland [23] a mismatch in the timing of peak parturition for caribou and the proportion of emergent forages was weakly correlated with reduced survival of offspring (but contrary to [16]). This reported trophic mismatch pertained specifically to a single component of peak nutrient availability (i.e., N concentration or forage quality). Similarly, survival to winter of roe deer (*Capreolus capreolus*) fawns declined with an increasing mismatch in the onset of the growing season and fawning date [24]. Offspring survival of muskoxen may have responded negatively to mismatches between the onset of the growing season and parturition in the year prior to birth [25]. In contrast, earlier springs and more vegetatively productive growing seasons increased reproductive success for reindeer in Fennoscandia [17] and survival of caribou neonates in Alaska [26], and recruitment in Svalbard reindeer was strongly related to late winter maternal mass not spring phenology [16].

Characteristics of the Arctic growing season have large influences on primary productivity, which in turn affect maternal body mass, parturition rate, maternal provisioning, and (or) offspring mass of large herbivores [27, 28]. Timing and magnitude of nutritional demands of parturient females vary with degree of reliance on maternal capital. Although reproductive investment occurs along a continuum from capital to income [6], the nutrient allocation data clearly demonstrate that for caribou [28, 29], as in other Arctic ungulates [8], the N source for reproduction is primarily from maternal capital. The nutritional demands of reproduction for females increase from birth to peak lactation, which occurs approximately three weeks after parturition [30]. However, the amount of maternal capital necessary for reproduction may be reduced by dietary income either through modification of milk production and (or) by forage availability to offspring. Subsequently, a female’s “investment” in her offspring rapidly declines as her offspring acquires progressively more nutrients from forages other than milk and increasingly abundant forage resources are preferentially deposited into her somatic tissues [29]. Indeed, the remainder of the growing season is marked by rapid mass gains in mother-offspring pairs. Evaluation of the mismatch hypothesis has focused primarily on parturition date [23] and less so on the nutritionally demanding period of early lactation [31]. Additionally, examinations of potential climate-induced trophic mismatches in other periods of the growing season (e.g., peak biomass in mid-summer and vegetative senescence in the autumn) that may influence mother-offspring mass have largely been overlooked (except for [32, 33]).

To advance the understanding of the climate-mediated forage influences on northern ungulates, we evaluated the trophic mismatch hypothesis as suggested for caribou [23]. Specifically, we examined whether a trophic mismatch occurred between resource availability (as indexed by N concentration in forages at the start of the growing season) and peak parturition and peak lactation. We compared the temporal changes in the window of seasonal plant growth with the quality of food available to a caribou herd with a consistent timing of parturition (1–7 Jun [34]). We evaluated the long-term changes in the temperatures, timing, and length of the growing seasons (1970–2013), and compared growing conditions and dynamics of forage quality (N concentration) in four important forages of caribou at peak parturition, peak lactation, peak forage biomass, and plant senescence between the growing seasons of 1977 and 2011–13 in three ecoregions in the Alaskan Arctic. Support for the trophic mismatch hypothesis (as stated in [23]) would require warming trends to result in 1) earlier initiation of plant growth and 2) decreased forage quality in 2011–13 compared to 1977 at peak parturition and (or) peak lactation on birthing grounds. Further, we used this investigation to discuss the match-mismatch framework and present additional hypothetical elements that may influence the potential climate-mediated effects on the nutrition of northern ungulates throughout the growing season.

## Materials and methods

We studied a 200-km transect spanning three Arctic ecoregions along the Dalton Highway on the North Slope of the Brooks Range in Alaska. These ecoregions are underlain with thick continuous permafrost and contain several north to south gradients in latitude (~4°), elevation (0–2,400 m), annual precipitation (140–260 mm), and vegetation. Although these ecoregions have similar average winter temperatures (November—March; approx. -24°C), summer temperatures (June-August) are quite different (2000–2009; Scenarios Network or Alaska and Arctic Planning, CRUTS3.1, http://www.snap.uaf.edu/data.php).

The Alaskan Arctic Coastal Plain ecoregion (hereafter the Coastal Plain) is bounded on the north and the west by the Arctic Ocean, on the east by the Alaskan-Yukon border and on the south by the Brooks Range foothills [35]. Current average daily temperature in summer is near 7°C. The Coastal Plain is a poorly drained, treeless plain covered by thaw lakes and wetland complexes interspersed with ice-wedge polygons and slightly acidic to neutral soils. The primary vegetative community is wet graminoid that is typically dominated by water sedge (*Carex aquatilis*) and tall cottongrass (*Eriophorum angustifolium)* with mosses and dwarf shrubs typically on hummocks. The Coastal Plain rises gradually from sea level to approximately 180 m in the Arctic Foothills to the south.

The Arctic Foothills ecoregion (hereafter the Foothills) is a mostly treeless band of plateaus and hills between the Coastal Plain and the Brooks Range [35]. Current average daily temperature in summer is 11°C with daytime maximum temperatures approaching, and sometimes exceeding, 25°C in late June. The hills and valleys are better drained with fewer lakes than the Coastal Plain. Predominant vascular species include tussock cottongrass (*E*. *vaginatum*), Bigelow’s sedge (*C*. *bigelow*), diamond-leaf willow (*Salix pulchra*), mountain cranberry (*Vaccinium vitis-idaea*), and dwarf birch (*Betula nana*) in the uplands; water sedges in the lowlands; as well dwarf scrub communities in drier or exposed sites at higher elevations near the boundary of the Northern Brooks Range ecoregion (approx. 425–600 m [36]).

The Northern Brooks Range ecoregion (hereafter the Brooks Range) consists of rugged and steep mountains (800–2,400 m) that span the east-west extents of northern Alaska [35]. Current average daily temperature in summer is 11°C with lower daily maximums than the Foothills. Unstable mountainsides are sparsely covered with dwarf scrub vegetation whereas mesic sites in the valleys are covered with graminoid herbaceous communities especially on the northern boundary along the Foothills. Erect shrub tussock tundra on acidic soils is dominated by willow and dwarf birch, tussock cottongrass, and other sedges (*Carex* spp.), typically Bigelow’s sedge, with mosses and fruticose lichens abundant in the understory [36].

The Central Arctic herd is the primary caribou population inhabiting this area. Since regular monitoring of this herd began in 1975, the Central Arctic herd increased from 5,000 to 70,000 by 2010 [37], but has recently declined to 22,000 (Alaska Department of Fish and Game, unpublished data). This herd typically migrates in May from the Brooks Range and calves in early June between the Colville and Canning Rivers on the Coastal Plain [38]. Peak parturition occurred during 1–7 June [34, 37]. As summer progresses, caribou may move south off the Coastal Plain towards the Foothills to seek out areas that minimize harassment by parasitic insects. Later, as plants senesce, animals move south to fall and winter ranges in the Foothills and along the northern and southern slopes of the Brooks Range [39].

### Broad scale characteristics of the growing season (1970–2013)

**Fig 1:**
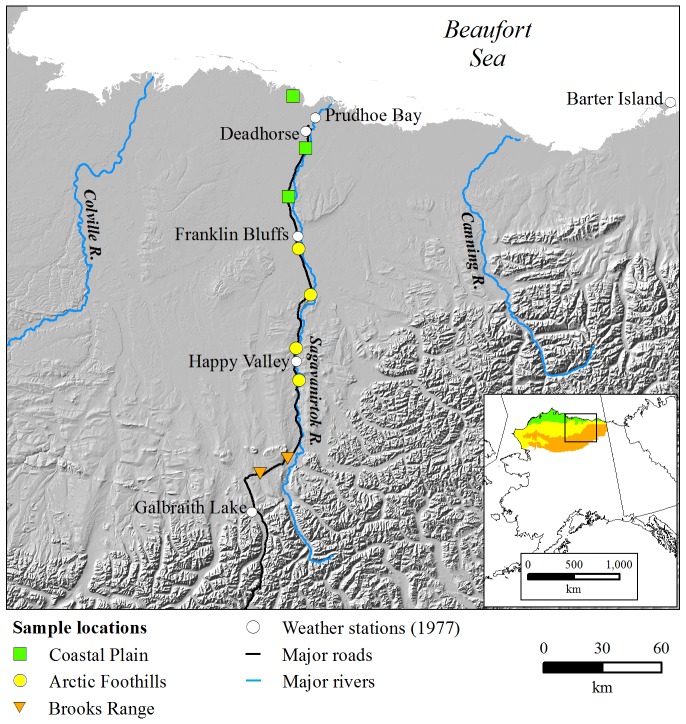
The locations of weather stations and sampling locations to monitor nitrogen content of caribou (*Rangifer tarandus*) forages and ambient air temperatures throughout the growing seasons of 1977 and 2011–13 in three ecoregions (Coastal Plain, Foothills, and Brooks Range) along the Dalton Highway, North Slope of the Brooks Range, Alaska; distribution of the Arctic ecoregions in Alaska are shown in the inset.

To document long-term trends in temperature and phenology of the growing season in the central Alaskan Arctic (Fig 1), we used average monthly temperatures from 1970–2013 to assess temperature trends and estimate day of thaw, day of freeze, and length of the growing season for each year and ecoregion. Monthly temperature data were extracted from subsets of each ecoregion along the Sagavanirktok River (approx. 80 km wide) from downscaled data sets derived from historical observations (1970–2009; CRUTS3.1; http://www.snap.uaf.edu/data.php) as well as observations from Natural Resources Conservation Service’s SNOTEL sites in northern Alaska [2000–2013; Prudhoe Bay (1177), Sagwon (1183), and Imnaviat Creek (968); http://www.wcc.nrcs.usda.gov/snotel/Alaska/alaska.html). Mean monthly temperatures were used to describe monthly temperature trends for May—September 1970–2013.

To estimate the day of thaw and freeze for each year and ecoregion, we regressed the monthly mean temperatures from January to June (day of thaw) and July to December (day of freeze); estimated the fractional month when monthly mean temperature = 0°C; and multiplied by 365.24 to estimate the day of the year when thaw (≥0°C) or freeze (≤0°C) occurred (similar to http://ckan.snap.uaf.edu/dataset/historical-derived-dof-dot-logs-771m-cru-ts). We assumed the length of the growing season was the day of thaw subtracted from the day of freeze for each year [40]. Linear regression, 95% confidence intervals of slopes, and coefficients of determination (*r*^2^) were used to evaluate trends in temperature and characteristics of growing seasons within each ecoregion since 1970 [41]. To evaluate temperatures and characteristics of the growing seasons in the years of forage sampling (1977 and 2011–13) relative to long-term trends, we used residuals of the regressions of temperature, days of thaw and freeze, and length of the growing season on year (1970–2013).

### Site specific growing conditions and forage quality (1977 and 2011–13)

To compare growing conditions at our sample sites between 1977 [42] and 2011–13 throughout the growing season (12 May—Sep 30), we used daily temperatures within each ecoregion from existing weather databases (1977) and data loggers at our 9 sampling locations (2011–2013; Fig 1). For 1977, we downloaded hourly temperature data (http://www.ncdc.noaa.gov/cdo-web/) from Barter Island (Coastal Plain), Prudhoe Bay (Coastal Plain), Deadhorse (Coastal Plain), Franklin Bluffs (Foothills), Happy Valley (Foothills), and Galbraith Lake (Brooks Range) and summarized hourly temperatures by day. Except for Barter Island, the availability of consecutive daily temperature data was poor for the Coastal Plain in 1977. Average daily temperatures at Barter Island throughout the growing seasons of 1973–1980 were strongly correlated to daily temperatures at Prudhoe Bay [*n* = 698; *r* = 0.95] and Deadhorse [*n* = 331; *r* = 0.94]. We, therefore, used data from Barter Island and the following equations to model missing temperatures for Prudhoe Bay and Deadhorse to estimate growing conditions in 1977 on the Coastal Plain (Fig 1): Prudhoe (average daily temperature °C) = 1.178*Barter Island (average daily temperature °C) + 1.772; Deadhorse (average daily temperature °C) = 1.283*Barter Island (average daily temperature °C) + 1.152.

In 2011–13, we used data loggers (UA-002-64; Onset Computer Corporations, Bourne, MA) housed in solar radiation shields (SRS100; Ambient Weather, Chandler, AZ) to monitor hourly temperatures (°C) at each sampling location. Loggers and shields were approximately 1 m above the ground on wooden dowels (3.2 cm in diameter); hourly temperatures for each plot were summarized by day and year. To index cumulative summer “warmth” or growing conditions for forages, average daily temperatures were converted to thaw degree days (thaw degree day is average daily temperature > 0°C) and summed to create cumulative thaw degree days curves by ecoregion for 1977 and 2011–13. In order to identify potential differences in temperature readings from sensors at existing weather stations and data loggers, we used linear regression to examine the correlation between daily mean temperatures at Deadhorse and data loggers in 2011–13. Temperatures recorded at nearby data loggers (<18 km) were strongly correlated to the Deadhorse station (*n* = 405, *r* = 0.97, slope ± 95CI = 1.0 ± 0.02; weather station data was also used as input in long-term datasets to evaluate characteristics of the growing season).

#### Collection and processing of forage samples

Similar to forage collections in 1977 [42], in 2011–13, we collected samples of current annual growth of 4 important foods for caribou [tussock cottongrass, water sedge, louseworts (*Pedicularis* spp.), and diamond-leaf willow] [43–45] approximately every 2 weeks (28 May–25 September) at 9 sampling locations along a 200-km transect. Representative samples (approx. 20–100 g) of forages were collected over large areas (approx. 5 ha) at approximately the same time (10^th^ and 25^th^ of each month ± 2.5 days) and locations [42]. In the field, we stored samples in paper bags in protected, shaded, and dry locations at ambient temperatures with constant air flow. Upon return from the field (≤6 days following collection), samples were dried in a forced-air oven at 50–55°C to constant mass [46], and stored at room temperature until processing. Samples were ground through a 1.27-mm screen in either a Wiley (Thomas Scientific, Swedesboro, New Jersey, USA) or centrifugal mill (Retsch ZM 200, Haan, Germany) and analyzed for total N content with an elemental analyzer (CNS2000; LECO, St. Joseph, MI, USA). Nitrogen content was expressed on the basis of dry matter content, which was determined by drying to constant mass at 80°C in a convection oven.

#### Nitrogen content by day of the year

Based on *a priori* knowledge from similar work on nitrogen dynamics in reindeer (*R*. *tarandus*) forages in Alaska [47, 48], we used regression to fit linear, quadratic, and cubic functions to estimate N content for each forage species on day of the year by ecoregion and period of collection (1977 and 2011−13). The large-scale changes in climate that occurred from 1970–2013 was considered the treatment, thus data in 1977 were considered control and 2011–13 the treatment. Additionally, exploratory (S1 and S2 Figs) and preliminary analyses (S1 Table; examined the main fixed effects of day of the year and year by ecoregion and species in 2011–13) supported pooling 2011–13 data, as year was not a significant factor in 8 of 12 models. Adjusted coefficients of determination (*r*^2^_a_ or *R*^2^_a_) were used to assess if additional polynomials improved the fit of the regression equation to the data, thus unlike coefficients of determination, additional parameters do not necessarily increase this measure of fit [41]. We used the best fit models and “predictnl” in Stata 11.2 (College Station, TX, USA) to estimate N content (± 95% confidence limits) by day of the year for each forage species and ecoregion throughout the growing seasons of 1977 and 2011–13. Model estimates were set according to the growing season’s earliest (Coastal Plain: day 164 in 2012; Foothills and Brooks Range: day 152 in 2012) and latest (all ecoregions: day 267 in 2012) days of the year that forage was sampled across all years of collection within each ecoregion and in some cases. For each forage species and ecoregion, we used 95% confidence intervals to compare N content between 1977 and 2011–13 for 4 important periods for the Central Arctic herd, including: 1) peak parturition (early June: days of year = 152−158 [34, 37]), 2) peak lactation (3 weeks after peak parturition: 173−179 [30]), 3) peak forage biomass (late July: 209−215; D. Gustine, unpublished data); and 4) plant senescence (the autumnal equinox; 261−267).

## Results

From 1970–2013, temperature trends for all ecoregions indicated warmer growing seasons, however, the strength of the warming trend was strongest on the Coastal Plain which is the birthing grounds of the Central Arctic caribou herd. Average temperatures increased in May (0.05°C·yr^-1^), June (0.04°C·yr^-1^), July (0.03°C·yr^-1^), and September (0.06°C·yr^-1^) on the Coastal Plain, while temperatures increased in June (0.03–0.04°C·yr^-1^) and September (0.04–0.05°C·yr^-1^) in the Foothills and Brooks Range (S2 Table).

**Fig 2:**
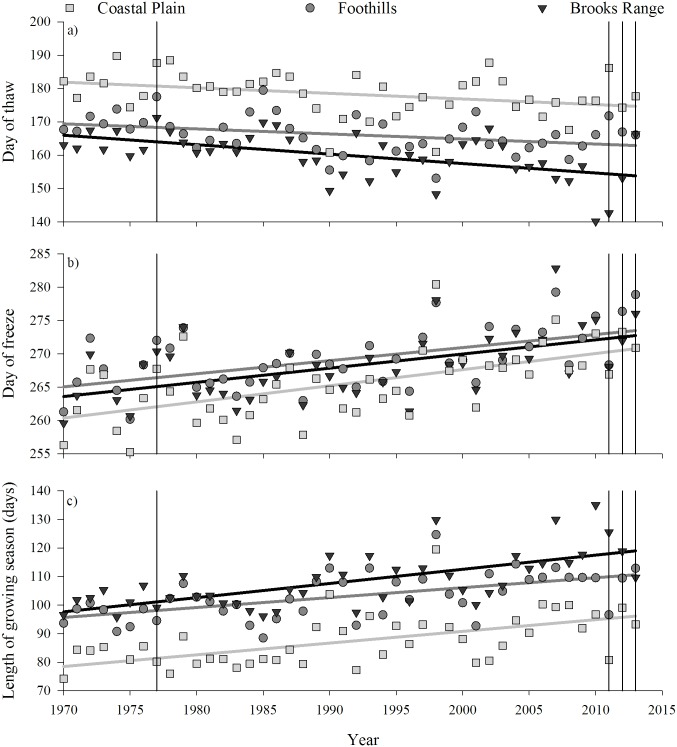
Estimates of the a) day of thaw, b) day of freeze, and c) length of the growing season in three ecoregions (Coastal Plain, Foothills, and Brooks Range) along the Dalton Highway, North Slope of the Brooks Range, Alaska; vertical lines denote years of forage sampling; and the confidence intervals (95%) of all slopes did not include zero (*r*^2^ = 0.11–0.44).

Recent growing seasons of all the ecoregions started 7−12 days earlier (Fig 2a), ended 9−10 days later (Fig 2b), and lasted 15−21 days longer than in 1970 (Fig 2c). In the Coastal Plain, growing seasons always started later and typically froze earlier than other ecoregions (Fig 2a and 2b). Growing seasons on the Coastal Plain were approximately 14−17 and 20−23 days shorter than the Foothills and Brooks Range, respectively (Fig 2c). In 1977, thaw occurred 7−9 days later across ecoregions than expected from the regression results. Indeed, the day of thaw in 1977 was the second latest day of thaw in the 44-year record (Fig 2a). There were very few similarities among growing season characteristics across ecoregions and years during 2011−13 (Fig 2). However, on the Coastal Plain in 2011−13, the days of thaw occurred 8 days earlier (average; range = 2–13 days) and the growing season was 11 days longer (0–20 days; Fig 2c) than in 1977.

**Fig 3:**
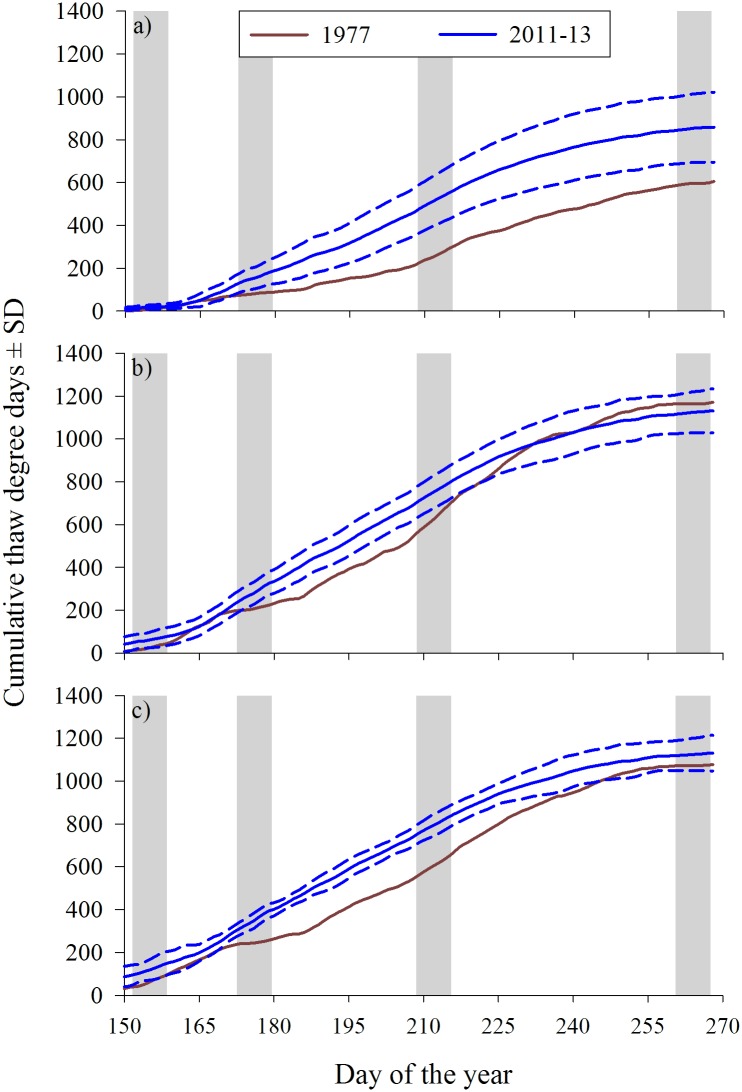
Growing conditions, as indexed by cumulative thaw degree days, throughout the growing seasons of 1977 and 2011–13 when caribou forages were collected within the a) Coastal Plain, b) Foothills, and the c) Brooks Range ecoregions on the North Slope of the Brooks Range in the Alaskan Arctic. Shading denotes the periods of interest (days of year): peak parturition (152–158), peak lactation (173–179), peak forage biomass (209–215), and senescence (261–267).

The growing seasons of 2011−13 were warmer than 1977 in the Coastal Plain and the Brooks Range. The Coastal Plain was cooler in 1977, as measured by cumulative thaw degrees, through peak calving and lactation [peak parturition (mean, range): 1977 = 8.8, 2.2–16.9 and 2011–13 = 17.6, 13.1–21.0; peak lactation: 1977 = 80.6, 73.6–86.5 and 2011–13 = 156.6, 132.7–181.7)]. Thermal differences between 1977 and 2011−13 were most pronounced at peak biomass in all ecoregions (Fig 3).

With a few exceptions, the best fit models to estimate the change in N content in forages throughout the growing seasons of 1977 (*n* = 153) and 2011−13 (*n* = 511) were curvilinear. Overall, models fit well for willow (mean *R*^2^_a_ = 0.91), and adequately for tussock cottongrass (0.69), water sedge (0.68), and louseworts (0.55; S3 Table).

**Fig 4:**
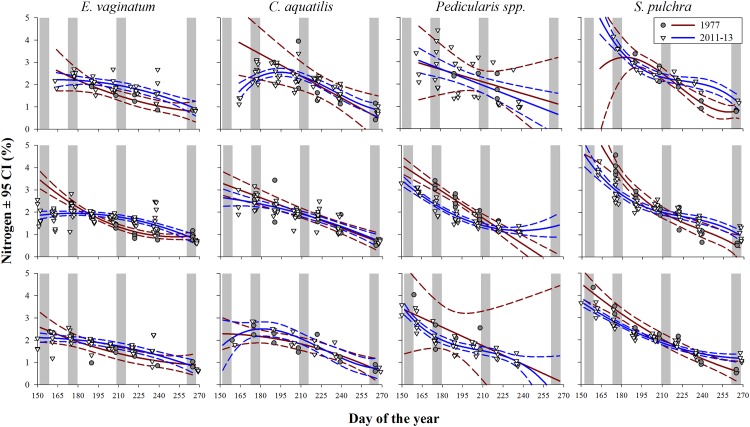
Nitrogen content (%) in four primary forages of caribou throughout the growing seasons of 1977 and 2011–13 within the Coastal Plain (top row), Foothills (middle row), and the Brooks Range (bottom row) ecoregions in the Alaskan Arctic; shading denotes the periods of interest (days of year): peak parturition (152–158), peak lactation (173–179), peak forage biomass (209–215), and senescence (261–267). Model estimates were limited to the earliest and latest dates that current annual growth were available for all periods within each ecoregion.

Forage quality (as indexed by N content) generally peaked at the beginning of the growing season and declined to senescence in all ecoregions (Fig 4; S5 Table). In contrast to the trophic mismatch prediction of decreased forage quality for fixed dates [23] in warmer 2011–2013 (S5 Table) compared to cooler 1977, there were no significant differences in N concentration during parturition or lactation. On the contrary, on the Coastal Plain where caribou gave birth, no emergent forages were even available for collection during peak parturition in either 1977 or 2011−13 (i.e., growing season had not started yet). In the Foothills, N concentration was higher in 1977 for cottongrass and water sedge at peak calving and lactation, however, this area was not typically used by caribou at these times.

## Discussion

Despite the advanced thaw date and increased growing season length (Figs 2 and 3) on the birthing grounds, the growing season had not commenced at peak parturition in 1977 or 2011–13 and we found no reduction in forage quality at peak lactation (Fig 4). Consequently, there was no support herein for the occurrence of a trophic mismatch (as defined in [23]) at the onset of the growing season for this population. The environmental conditions for a trophic mismatch in forage quality to occur were clearly in place, however, counter to observations in Greenland [23] but similar to elsewhere [16, 17, 49], key emergent forages were not available to reproductive females until approximately 4–11 days after peak parturition (Fig 4). Thus a “mismatch” in the onset of the growing season and reproductive phenology appears to be the norm for caribou in the Alaskan Arctic. Similarly, there has been no temporal trend in the timing of peak N concentration in forages or reproductive phenology of other *Rangifer* sp. populations in Fennoscandia [16, 17] as well as other vertebrates in the North American Arctic [14].

Arctic ungulates have committed maternal capital by giving birth before income is available as new plant growth. The resources required by Arctic ungulates to complete reproduction are established in the previous summer and fall [29]. The heterogeneity of Arctic systems at peak parturition selects for a capital breeding strategy in large mammals that must use large areas to complete their life history [50]. The responses of large herbivores may vary widely with the responses of Arctic plants to climate change because productivity of populations is tied to plant production through the entire growing season not just at the onset.

### Growing seasons

As has been typical of northern systems, the Alaskan Arctic experienced increased temperatures and advances in the phenology of growing seasons over the last 44 years. Trends in temperature and characteristics of the growing seasons were indicative of a strong warming signal: growing seasons were warmer, started earlier, ended later, and lasted longer (S2 Table and Fig 2). Even though Alaska apparently “cooled” in the first decade of the 21st century, northern Alaska has undergone a distinct warming trend in the summer months since the late 1970s [51]. Increases in temperature (S1 Table) were consistent with longer-term trends reported for the region [52]. Satellite-based indices of vegetative phenology and soil thaw corroborated our estimates suggesting growing seasons in the north start 4–17 days earlier than in the 1980s [53–55]. Further, modeling efforts estimated that leaf out started approximately 10 days earlier in 2013 than in 1970 [56]. Our estimates of changes in the length of the growing season from 1970 (0.37 days·yr^-1^) were similar to those reported in the Canadian Arctic (0.41 days·yr^-1^ [14]), as well as from a biome-level assessment of soil temperatures in the North American tundra (0.42 days·yr^-1^ [55]), but shorter than estimates in Svalbard (0.58 days·yr^-1^ [57]) or Greenland (0.77 days·yr^-1^ [58]). Although longer growing seasons may not necessarily mean warmer growing seasons (Fig 2b), growing seasons on the birthing grounds (i.e., Coastal Plain) started earlier (Fig 2a) and were 42% warmer in 2011–13 than 1977 (Fig 3a), similar to the 37% increase observed over a 22-year time series in the Canadian Arctic [14].

### Reproductive investment and trophic mismatch

Reproductive investment strategies can magnify (income breeders) or dampen (capital breeders) the consequences of a mismatch in peak resource demand and availability [7]. For income breeders, which predominantly rely on forage resources in late gestation and lactation, mismatches between reproductive and vegetative phenology may reduce fitness and population growth rates [24]. Conversely, the maternal reserves of capital breeders are deposited late in the previous growing season and are used to offset limited food availability in early reproduction. Therefore, capital breeders may experience weaker trophic feedbacks [7] to the timing of food availability and, possibly, a lag in the effect of a mismatch. Capital breeders, which include muskoxen [8, 25], Soay sheep (*Ovis aries*) [7], and *Rangifer* sp. [17, 28, 29], rely heavily on maternal reserves throughout reproduction and are robust to trophic mismatches at parturition and during lactation [16]. Similar to other circumpolar *Rangifer* populations [17, 28, 49, 59, 60], in the central Alaskan arctic, peak parturition was at least one week prior to the availability of spring re-growth of forages (Fig 4), and females likely depended heavily on maternal capital to support their offspring early in the growing season. Despite several decades of increasing temperatures (S1 Table; Fig 2) and dynamic population changes, the timing of peak parturition has remained consistent for northern Alaskan caribou populations [37, 61–63]. Meanwhile (as recently summarized in Veiberg et al [16]), caribou responses in western Greenland were similarly diverse: there was wide spatial variation in productivity of caribou in the West Greenland, which included the observations reported in Post and Forchhammer [23], when populations were stable or increasing [64–66].

Recruitment in capital breeders is not influenced by forage quality (N content) alone but by the combination of quality and quantity throughout the growing season and its effect on the dynamics of maternal capital and offspring growth. Spatio-temporal variation in forage characteristics and foraging conditions [67, 68] induce a capital reproductive investment strategy (in sensu [28]). Forage abundance has large effects on the capacity of mothers to replenish and establish body stores [17, 69], thus recruitment is indirectly affected by environmental variation through changes in maternal reserves [7, 16]. However, dynamics of forage quality interact strongly with forage abundance to dictate timing of peak resource availability and, thereby, rates of nutrient gain. Therefore, we suggest that to effectively evaluate the mismatch hypothesis [70] it is crucial to determine the timing of peak resource demand and peak resource availability relative to the investment strategy of reproduction of the species of interest [10]. For example, in caribou, peak N demands of lactation occur approximately 3 weeks after parturition and are met primarily with body reserves but are offset with intake of emergent forages [29, 30]. After this period, lactating females rapidly shift their resource allocation to somatic tissues rather than offspring with protein deposited preferentially to fat [71]. Peak resource availability is a product of forage biomass, N concentration, and digestibility. Although the onset of the growing season in the Arctic typically coincides with peak N content (Fig 4), emergent forage biomass is very low but highly digestible at this time [72, 73]. Similar to the Canadian Arctic [74], peak forage N availability (e.g., kg digestible N · ha^-1^) on the Coastal Plain likely occurs after the peak demands of lactation but before the peak of forage biomass (Fig 4; D. Gustine, unpublished data) [75]. This coincides with a decline in milk intake by offspring [30] while forage intakes and body masses are increasing for both mothers and their offspring in direct response to increases in forage abundance. Body mass gains during this period for mother-offspring pairs are critical for conception in adult females [34] and overwinter survival and recruitment [16]. Therefore, complementary to Veiberg et al [16], we suggest the appropriate window of time to examine the match-mismatch framework in Arctic ungulates is not at parturition but in late summer-autumn, where the multiplier effects of small changes in forage quality [76] are amplified by forage abundance, peak forage intake, and resultant mass gains in mother-offspring pairs.

Foraging windows [10] for Arctic herbivores may shift with the spatial and temporal diversity of terrain, vegetation, and phenology. Although the onset of the growing season has typically occurred earlier as a result of warming (Fig 2a) [13], the direction and magnitude of the effect of vegetative responses to warming has varied across the Arctic [14, 15]. Phenological trends in vegetation in the Arctic are site dependent [13], thus phenological responses to warming are most likely heterogeneous across space and time [14, 15]. Responses are dynamic within forage groups as warmer springs in the high Arctic may increase N concentration in some sedge species, while warmer summers may increase the rate of lignification in others [14]. Inter-annual variation in forage abundance has also been estimated to be both low and high in response to temperatures [77, 78], while timing of peak forage abundance has typically remained static despite increases in temperature in the Arctic growing seasons [77]. Nevertheless, climate shifts are expected to continue to substantially increase vegetative biomass in Arctic systems, with most of the change due to the expansion of shrubs [79]. These shifts in temperature regimes, habitat composition, and forage abundance and quality will hypothetically affect the duration of foraging windows. Thus, we expect that heterogeneous vegetative responses to climate in spatially and phenologically diverse environments will spur similarly diverse responses among populations of caribou and other Arctic herbivores.

### Conclusions

Despite earlier thaw dates and warmer temperatures, we did not observe an advance in the timing of peak N concentration (i.e., forage quality) as expected under the trophic mismatch hypothesis (in sensu [23]). Evidence herein suggests that trophic mismatches for caribou in Alaska were not likely at parturition because caribou appear to use maternal capital to buffer lack of green forages at parturition and (or) temporal variance in forage quality and abundance [7, 28, 80]. Thus, the generalizability of the spring trophic mismatch hypothesis may be limited for caribou populations [16]. Rather, climatic influences on peak resource availability during the period of rapid forage intakes and mass gains (i.e., late summer to fall) may have stronger effects on reproductive success than maternal mismatch with forage quality alone at parturition. Independent of insect harassment, we hypothesize that earlier springs, increases in forage biomass [17, 26], longer growing seasons, and shifts in forage quality in summer-autumn ranges will provide nutritional benefits throughout the growing season to reproductive females [16]. However, given the wide spatial variation in shifts of phenology, abundance, and quality of forage; the capacity of migratory caribou to shift birthing and summering ranges [81] and to forage selectively and rapidly [82]; the marked behavioral and physiological plasticity evident in *Rangifer*; as well as the diversity of circumpolar habitats occupied by this species [83], we expect a diverse response among populations of caribou to continued climate warming.

## Supporting information

S1 Table Preliminary analyses used to determine whether data on nitrogen content (%) in the primary summer forages of caribou [*Rangifer tarandus*; tussock cottongrass (*Eriophorum vaginatum*), water sedge (*Carex aquatilis*), louseworts (*Pedicularis* spp.), and diamond-leaf willow (*Salix pulchra*)] in 3 ecoregions on the North Slope of the Brooks Range, Alaska could be pooled across years for 2011–13. Models used analysis of covariance to examine fixed main effects of day of the year and year on nitrogen content by ecoregion and species.
(DOCX)

S2 Table The trends in temperature for each period of the growing season and ecoregion from 1970–2013 (*n* = 44 years) as well as the deviations from the long-term trends in temperature for the years forages were sampled on the North Slope of the Brooks Range, Alaska in 1977 and 2011–13. Slopes (β) with 95% confidence intervals that did not include zero are denoted in bold.
(DOCX)

S3 Table Linear and curvilinear models to estimate nitrogen content (%) from day of the year (doy) for the primary summer forages of caribou [*Rangifer tarandus*; tussock cottongrass (*Eriophorum vaginatum*), water sedge (*Carex aquatilis*), louseworts (*Pedicularis* spp.), and diamond-leaf willow (*Salix pulchra*)] in 3 ecoregions on the North Slope of the Brooks Range, Alaska in 1977 and 2011–13. (DOCX)

S4 Table The nitrogen (N) content (% of dry matter) for the primary summer forages of caribou [*Rangifer tarandus*; tussock cottongrass (*Eriophorum vaginatum*: erva), water sedge (*Carex aquatilis*: caaq), louseworts (*Pedicularis* spp.: pela), and diamond-leaf willow (*Salix pulchra*: sapu)] by day of the year and plot in Brooks Range (BR), Arctic Foothills (AF), and Coastal Plain (CP) ecoregions on the North Slope of the Brooks Range, Alaska in 1977 and 2011–13. (XLSX)

S5 Table The daily temperature data (minimum, mean, and maximum and cumulative thaw degree days) by year, day of the year, ecoregion, and plot along a south-north transect (Fig 1) on the North Slope of the Brooks Range, Alaska 2011–13. (XLSX)

S1 Fig Predicted (2011–13) and observed nitrogen content (%) by year in four primary forages of caribou throughout the growing seasons of within the Coastal Plain (top row), Foothills (middle row), and the Brooks Range (bottom row) ecoregions in the Alaskan Arctic; shading denotes the periods of interest (days of year): peak parturition (152–158), peak lactation (173–179), peak forage biomass (209–215), and senescence (261–267). Model estimates were limited to the earliest and latest dates that current annual growth were available for all periods within each ecoregion.
(TIF)

S2 Fig Relationship between the predicted (Fig 4) and observed nitrogen content (%) by year in four primary forages of caribou on North Slope of the Brooks Range, Alaska, 2011–13. (TIF)
