# Supplementary material for: Advancing the match-mismatch framework for large herbivores in the Arctic: Evaluating the evidence for a trophic mismatch in caribou
Source: PLoS One. 2017 Feb 23;12(2):e0171807. doi: 10.1371/journal.pone.0171807 (PMC5322966; doi:10.1371/journal.pone.0171807)
Supplement: S1 Table — Models used analysis of covariance to examine fixed main effects of day of the year and year on nitrogen content by ecoregion and species. (DOCX) [file pone.0171807.s001.docx]

| Table S1. Preliminary analyses used to determine whether data on nitrogen content (%) in the primary summer forages of caribou [*Rangifer tarandus*; tussock cottongrass (*Eriophorum vaginatum*), water sedge (*Carex aquatilis*), louseworts (*Pedicularis* spp.), and diamond-leaf willow (*Salix pulchra*)] in 3 ecoregions on the North Slope of the Brooks Range, Alaska could be pooled across years for 2011–13. Models used analysis of covariance to examine fixed main effects of day of the year and year on nitrogen content by ecoregion and species. | | | | | | | | |
| --- | --- | --- | --- | --- | --- | --- | --- | --- |
| Ecoregion | Species | *n^a^* | *R*^2^_a_^b^ | Day of the year | |  | Year | |
|  |  |  |  | *F* | *P*-value |  | *F* | *P*-value |
| Coastal Plain | *E. vaginatum* | 37 | 0.56 | 37.00 | <0.001 |  | 2.83 | 0.073 |
|  | *C. aquatilis* | 54 | 0.43 | 37.94 | <0.001 |  | 3.77 | 0.030 |
|  | *Pedicularis* spp. | 30 | 0.51 | 16.4 | <0.001 |  | 6.86 | 0.004 |
|  | *S. pulchra* | 17 | 0.86 | 101.58 | <0.001 |  | 1.61 | 0.237 |
| Foothills | *E. vaginatum* | 85 | 0.42 | 52.70 | <0.001 |  | 2.22 | 0.115 |
|  | *C. aquatilis* | 52 | 0.69 | 116.75 | <0.001 |  | 1.07 | 0.351 |
|  | *Pedicularis* spp. | 42 | 0.85 | 201.79 | <0.001 |  | 5.20 | 0.010 |
|  | *S. pulchra* | 54 | 0.82 | 233.87 | <0.001 |  | 4.33 | 0.018 |
| Brooks Range | *E. vaginatum* | 46 | 0.54 | 47.18 | <0.001 |  | 2.21 | 0.123 |
|  | *C. aquatilis* | 16 | 0.67 | 116.75 | <0.001 |  | 1.07 | 0.351 |
|  | *Pedicularis* spp. | 38 | 0.79 | 129.78 | <0.001 |  | 2.27 | 0.119 |
|  | *S. pulchra* | 40 | 0.87 | 245.41 | <0.001 |  | 1.64 | 0.208 |
| ^a^Sample size | | | | | | | | |
| ^b^Adjusted coefficient of determination (Zar 1999) | | | | | | | | |
