# Supplementary material for: Advancing the match-mismatch framework for large herbivores in the Arctic: Evaluating the evidence for a trophic mismatch in caribou
Source: PLoS One. 2017 Feb 23;12(2):e0171807. doi: 10.1371/journal.pone.0171807 (PMC5322966; doi:10.1371/journal.pone.0171807)
Supplement: S2 Table — Slopes (β) with 95% confidence intervals that did not include zero are denoted in bold. (DOCX) [file pone.0171807.s002.docx]

| Table S2. The trends in temperature for each period of the growing season and ecoregion from 1970–2013 (*n* = 44 years) as well as the deviations from the long-term trends in temperature for the years forages were sampled on the North Slope of the Brooks Range, Alaska in 1977 and 2011–13. Slopes (β) with 95% confidence intervals that did not include zero are denoted in bold. | | | | | | | | | | | |
| --- | --- | --- | --- | --- | --- | --- | --- | --- | --- | --- | --- |
| Ecoregion | Period | Year | | | | |  | Residuals for years forages were sampled | | | |
|  |  | β^a^ | SE^b^ | Intercept | SE | *r*^2^ |  | 1977 | 2011 | 2012 | 2013 |
| Coastal Plain | May | **0.049** | 0.019 | -103.0 | 37.02 | 0.14 |  | 0.02 | 0.05 | 0.10 | -1.44 |
|  | June | **0.035** | 0.011 | -64.6 | 21.25 | 0.20 |  | 0.04 | -2.04 | 0.53 | 0.89 |
|  | July | **0.033** | 0.011 | -56.9 | 22.48 | 0.17 |  | -0.20 | -0.83 | 0.74 | -0.59 |
|  | August | 0.034 | 0.018 | -61.4 | 36.83 | 0.08 |  | 3.09 | -1.17 | 1.79 | -0.44 |
|  | September | **0.063** | 0.021 | -124.0 | 41.36 | 0.18 |  | 2.69 | 0.65 | 1.39 | -1.77 |
| Foothills | May | 0.023 | 0.02 | -48.1 | 39.47 | 0.03 |  | -0.17 | 0.35 | -0.77 | -2.20 |
|  | June | **0.030** | 0.012 | -50.3 | 23.47 | 0.13 |  | -0.04 | -2.64 | -0.27 | 1.10 |
|  | July | 0.014 | 0.013 | -15.3 | 25.55 | 0.03 |  | 0.61 | -1.86 | 0.52 | -1.39 |
|  | August | 0.007 | 0.016 | -5.3 | 32.24 | 0.01 |  | 2.85 | -1.40 | 0.79 | -1.32 |
|  | September | **0.046** | 0.019 | -90.0 | 38.41 | 0.12 |  | 1.98 | -0.59 | 0.47 | -1.88 |
| Brooks Range | May | 0.032 | 0.023 | -64.7 | 45.83 | 0.04 |  | -0.11 | 2.19 | -1.74 | -4.77 |
|  | June | **0.039** | 0.01 | -69.2 | 19.62 | 0.28 |  | 0.25 | 0.81 | 0.57 | -0.27 |
|  | July | 0.004 | 0.013 | 3.3 | 25.04 | 0.01 |  | 0.24 | -1.81 | -0.91 | -1.42 |
|  | August | -0.010 | 0.018 | 30.3 | 34.92 | 0.01 |  | 2.58 | -1.97 | -1.56 | -2.85 |
|  | September | **0.041** | 0.021 | -81.4 | 40.86 | 0.09 |  | 2.12 | -0.48 | -0.62 | -2.56 |
| ^a^Slope of the linear regression of temperature on year | | | | | | | | | | | |
| ^b^Standard error | | | | | | | | | | | |
