# Supplementary material for: Advancing the match-mismatch framework for large herbivores in the Arctic: Evaluating the evidence for a trophic mismatch in caribou
Source: PLoS One. 2017 Feb 23;12(2):e0171807. doi: 10.1371/journal.pone.0171807 (PMC5322966; doi:10.1371/journal.pone.0171807)
Supplement: S3 Table — (DOCX) [file pone.0171807.s003.docx]

| TableS3. Linear and curvilinear models to estimate nitrogen content (%) from day of the year (doy) for the primary summer forages of caribou [*Rangifer tarandus*; tussock cottongrass (*Eriophorum vaginatum*), water sedge (*Carex aquatilis*), louseworts (*Pedicularis* spp.), and diamond-leaf willow (*Salix pulchra*)] in 3 ecoregions on the North Slope of the Brooks Range, Alaska in 1977 and 2011–13. | | | | | | | | |
| --- | --- | --- | --- | --- | --- | --- | --- | --- |
| Ecoregion | Species | Year | *n*^a^ | *r*^2^_a_ or *R*^2^_a_*^b^* | Model terms | | | |
|  |  |  |  |  | doy | doy^2^ | doy^3^ | Intercept |
| Coastal Plain | *E. vaginatum* | 1977 | 7 | 0.87 | -0.068778658 | 0.000117848 |  | 10.75474531 |
|  |  | 2011–13 | 37 | 0.55 | 0.042133504 | -0.000126864 |  | -1.280148853 |
|  | *C. aquatilis* | 1977 | 10 | 0.55 | -0.032856340 |  |  | 9.283245485 |
|  |  | 2011–13 | 54 | 0.64 | 1.018763745 | -0.004471242 | 0.000006342 | -73.10881684 |
|  | *Pedicularis* spp. | 1977 | 6 | 0.17 | -0.017861787 |  |  | 5.878113442 |
|  |  | 2011–13 | 30 | 0.30 | -0.023464275 |  |  | 6.907650514 |
|  | *S. pulchra* | 1977 | 13 | 0.93 | 1.546279253 | -0.007216445 | 0.000010888 | -104.817937 |
|  |  | 2011–13 | 17 | 0.92 | -1.476499121 | 0.006493207 | -0.000009571 | 114.6691845 |
| Foothills | *E. vaginatum* | 1977 | 22 | 0.91 | -0.127910285 | 0.000253844 |  | 17.00949887 |
|  |  | 2011–13 | 85 | 0.52 | 0.050093046 | -0.000142124 |  | -2.464229048 |
|  | *C. aquatilis* | 1977 | 17 | 0.73 | -0.022690183 |  |  | 6.720756442 |
|  |  | 2011–13 | 52 | 0.70 | 0.014826959 | -0.000075739 |  | 2.144242356 |
|  | *Pedicularis* spp. | 1977 | 14 | 0.90 | -0.039565996 |  |  | 10.06304044 |
|  |  | 2011–13 | 42 | 0.88 | -0.139035524 | 0.000291784 |  | 17.73898428 |
|  | *S. pulchra* | 1977 | 20 | 0.92 | -0.937730260 | 0.003859069 | -0.000005427 | 78.99002117 |
|  |  | 2011–13 | 54 | 0.85 | -0.761996899 | 0.003332576 | -0.000004931 | 60.7204533 |
| Brooks Range | *E. vaginatum* | 1977 | 12 | 0.70 | -0.055337177 | 0.000097232 |  | 8.738153542 |
|  |  | 2011–13 | 46 | 0.56 | 0.026342313 | -0.000090407 |  | 0.196450613 |
|  | *C. aquatilis* | 1977 | 13 | 0.71 | 0.033116543 | -0.000113231 |  | -0.127042173 |
|  |  | 2011–13 | 16 | 0.75 | 0.758409934 | -0.003494641 | 0.000005162 | -50.88889681 |
|  | *Pedicularis* spp. | 1977 | 6 | 0.20 | -0.027811408 |  |  | 7.591619964 |
|  |  | 2011–13 | 38 | 0.85 | -0.959119278 | 0.004594137 | -0.000007424 | 69.08831645 |
|  | *S. pulchra* | 1977 | 13 | 0.93 | -0.089647817 | 0.000133596 |  | 14.97146747 |
|  |  | 2011–13 | 40 | 0.92 | -0.088063556 | 0.000159248 |  | 13.33699551 |
| ^a^Sample size | | | | | | | | |
| ^b^Adjusted coefficient of determination (Zar 1999) | | | | | | | | |
