# Supplementary figures and images for: Advancing the match-mismatch framework for large herbivores in the Arctic: Evaluating the evidence for a trophic mismatch in caribou
Source: PLoS One. 2017 Feb 23;12(2):e0171807. doi: 10.1371/journal.pone.0171807 (PMC5322966; doi:10.1371/journal.pone.0171807)

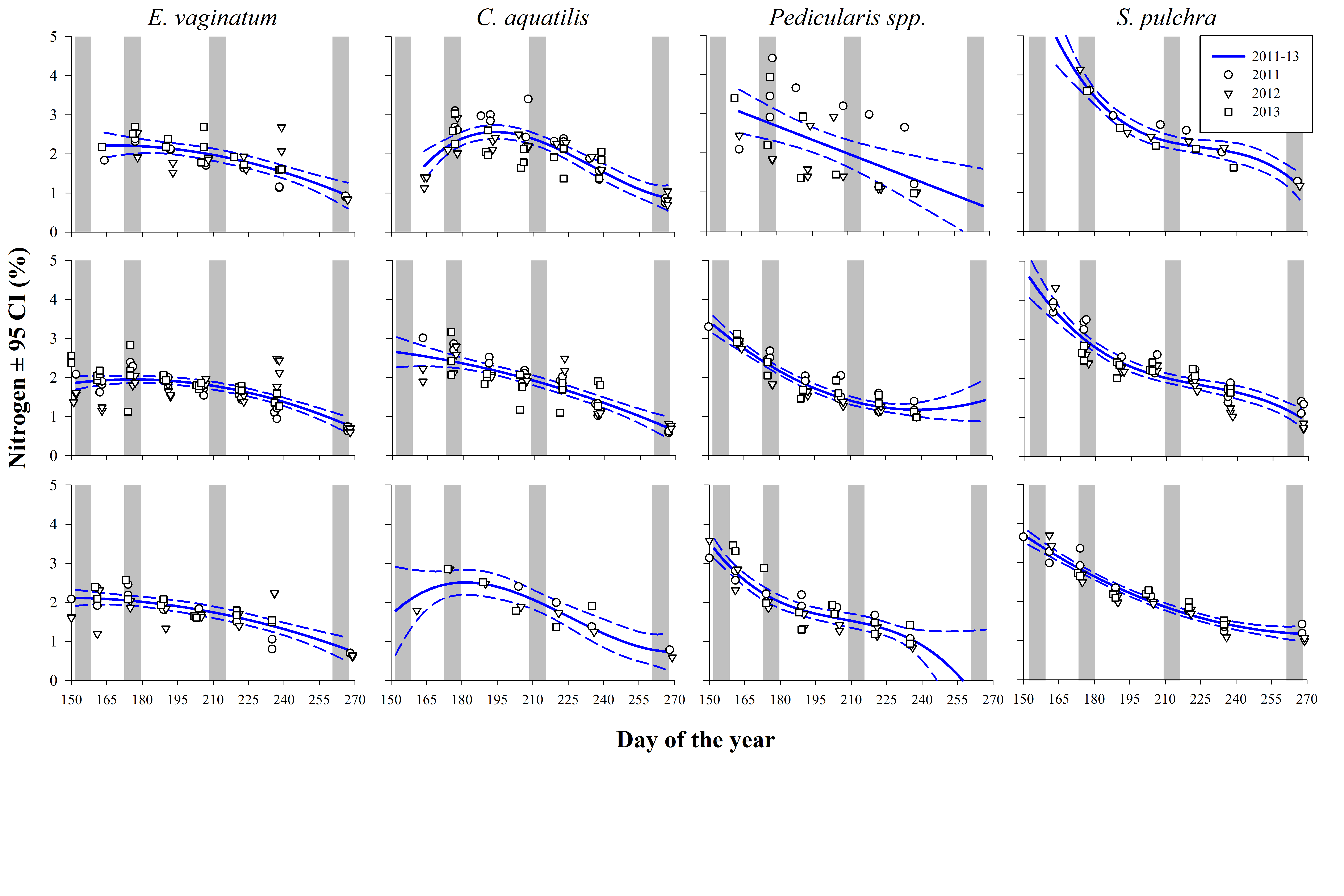

Supplement: S1 Fig — Model estimates were limited to the earliest and latest dates that current annual growth were available for all periods within each ecoregion. (TIF) [file pone.0171807.s006.tif]

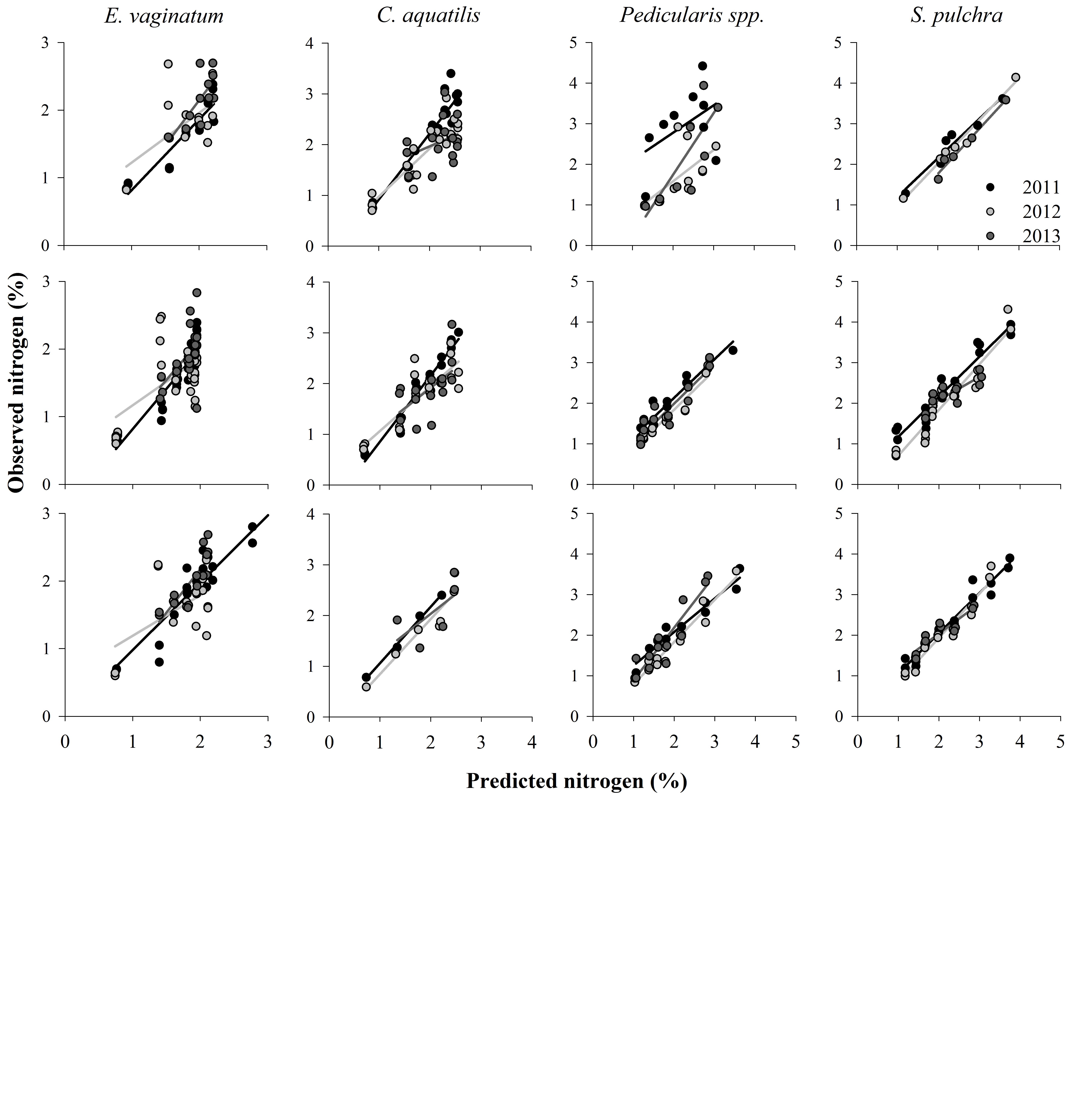

Supplement: S2 Fig — (TIF) [file pone.0171807.s007.tif]
